# Resolved Central Serous Chorioretinopathy Mimicking Hydroxychloroquine Toxicity: A Case Series and Literature Review

**DOI:** 10.3390/diagnostics15172154

**Published:** 2025-08-26

**Authors:** Seong Joon Ahn

**Affiliations:** Department of Ophthalmology, Hanyang University Hospital, Hanyang University College of Medicine, Seoul 04763, Republic of Korea; ahnsj81@gmail.com; Tel.: +82-2-2290-8574

**Keywords:** central serous chorioretinopathy, differential diagnosis, hydroxychloroquine retinopathy

## Abstract

**Background and Clinical Significance**: Central serous chorioretinopathy (CSCR) and hydroxychloroquine (HCQ) retinopathy can both cause outer retinal changes in systemic lupus erythematosus (SLE) patients treated with HCQ and corticosteroids. Differentiating between transient steroid-induced CSCR and irreversible HCQ toxicity is critical to avoid unnecessary discontinuation of essential therapy. **Case Presentation**: Three female SLE patients (ages 47, 41, and 37) on long-term HCQ (25, 9, and 6 years, respectively) and recent or ongoing low-dose prednisolone presented with unilateral OCT findings, parafoveal or pericentral photoreceptor defects, with the fellow eye unaffected. Review of clinical history and serial imaging revealed transient subretinal fluid in all cases, associated with recent corticosteroid use or dose escalation. Subsequent tapering or cessation of steroids led to resolution of the fluid, and earlier OCT scans confirmed normal outer retinal morphology, indicating that these changes were residual effects of resolved CSCR rather than HCQ toxicity. In Cases 1 and 2, the best-corrected visual acuity (BCVA) in the affected eye declined from 20/22 to 20/40 during the CSCR episode and improved to 20/30 and 20/25, respectively, after subretinal fluid resolution. In Case 3, by contrast, BCVA remained stable at 20/20 throughout the pre-, during-, and post-CSCR periods. **Conclusions**: Resolved CSCR can mimic HCQ retinopathy. These cases emphasize the importance of detailed medication history, serial multimodal retinal imaging, and comparison with prior and fellow-eye scans to distinguish resolved CSCR from HCQ retinopathy. Such thorough evaluation and careful differential diagnosis help ensure appropriate management—avoiding unnecessary HCQ discontinuation while protecting both ocular and systemic health.

## 1. Introduction

Central serous chorioretinopathy (CSCR) is an ophthalmologic condition characterized by the pathological accumulation of serous fluid beneath the neurosensory retina [1]. This fluid originates from the choroid due to a dysfunctional retinal pigment epithelium (RPE), leading to a small detachment and visual distortion [2]. Patients typically present with acute symptoms such as decreased visual acuity, metamorphopsia, a central scotoma, and diminished contrast and color sensitivity [2]. Clinical examination, typically with optical coherence tomography (OCT), reveals a serous retinal detachment [1,2]. Pigment epithelial detachments (PEDs), RPE mottling and atrophy, and subretinal fibrin may also be observed, depending on the chronicity. The annual incidence of CSCR in higher-income countries has been reported to range from 10 to 50 per 100,000 in men and from 2 to 16 per 100,000 in women, with the highest prevalence seen in middle-aged males [3,4]. In lower-income countries, data are more limited; however, restricted access to retinal specialists increases the likelihood of misdiagnosis [5], as non-retinal ophthalmologists may more readily misinterpret CSCR and its sequelae as other maculopathies.

A well-established and significant risk factor for the development or exacerbation of CSCR is corticosteroid use, irrespective of the route of administration (oral, topical, inhaled, or injected) [1]. Clinical studies have consistently demonstrated a strong association, with a notable proportion of CSCR patients reporting corticosteroid use shortly before presentation [6,7]. The mechanism is thought to involve inappropriate activation of mineralocorticoid pathways in ocular tissues, leading to choroidal vasodilation, vascular hyperpermeability, and subsequent RPE dysfunction [1,2].

Hydroxychloroquine (HCQ) retinopathy is a severe, often irreversible, and progressive toxic maculopathy that typically results from long-term HCQ therapy [8,9,10]. Although the underlying mechanism remains unclear, it is characterized by photoreceptor damage—with or without RPE involvement—primarily affecting the outer retina. One classic manifestation is bull’s-eye maculopathy, defined by a ring of parafoveal RPE depigmentation surrounding a spared foveal center; however, this presentation has become less common due to early detection through modern retinal imaging and standardized screening practices [10].

Systemic lupus erythematosus (SLE) is a complex chronic autoimmune disease affecting multiple organ systems [11], and ocular complications are reported in up to one-third of affected individuals [12,13]. These ocular manifestations can stem directly from the disease itself (e.g., lupus retinopathy, choroidopathy, or even CSCR) or as adverse effects of systemic therapies, notably corticosteroids and HCQ [14]. The convergence of SLE, corticosteroid use, and long-term HCQ therapy creates a particularly intricate clinical scenario. Macular changes in these patients can arise from any of these factors, often presenting with similar symptoms. This diagnostic ambiguity underscores the necessity for a meticulous differential diagnosis. A misdiagnosis could lead to the inappropriate cessation of HCQ, a cornerstone therapy for SLE management, potentially precipitating disease flares and increased systemic morbidity [15,16,17].

This case series serves to highlight a diagnostic dilemma encountered in clinical practice, particularly within the rheumatology–ophthalmology interface. This case series, with resolved CSCR mimicking HCQ toxicity, emphasizes the indispensable role of comprehensive patient history and serial multimodal imaging, especially historical data, in achieving an accurate diagnosis and ensuring optimal patient care.

## 2. Case Series Description

### 2.1. Case 1

**Figure 1:**
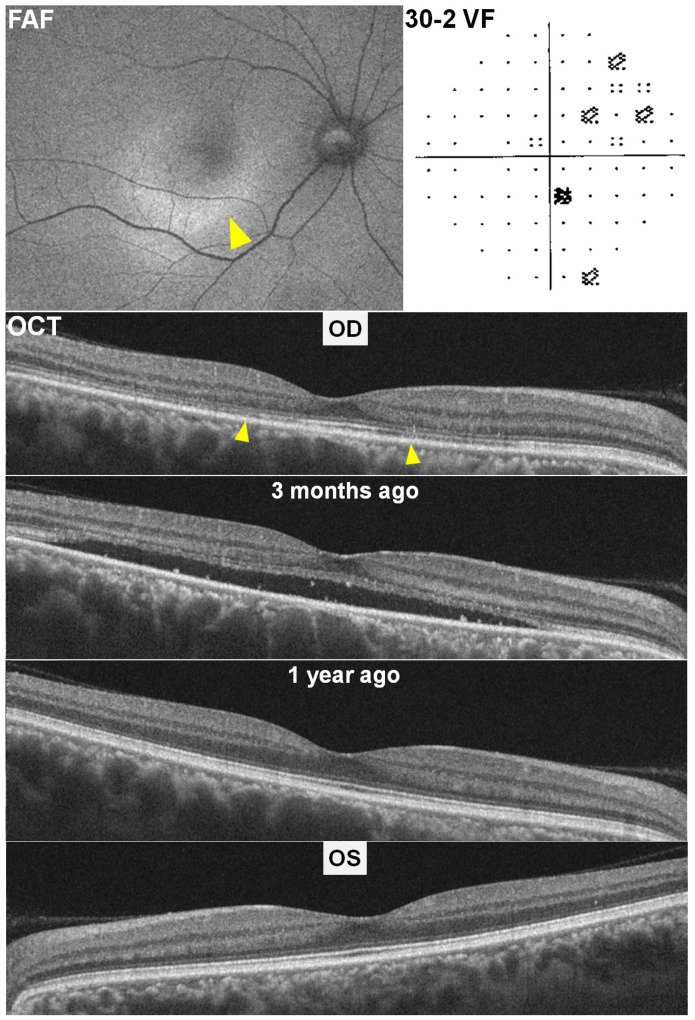
Multimodal imaging of Case 1 in the right (left column) and left (right column) eyes. Top row: Fundus autofluorescence (FAF) of the right eye shows ring-shaped inferior hyperautofluorescence (arrowhead), and 30-2 Humphrey visual field (30-2 VF) demonstrates scattered paracentral scotomas. Bottom row: Swept-source optical coherence tomography (OCT) of the right eye reveals parafoveal ellipsoid zone attenuation (arrowheads). OCT obtained three months earlier shows serous macular detachment (middle), which was absent on the prior annual OCT (bottom), supporting a diagnosis of steroid-induced central serous chorioretinopathy rather than hydroxychloroquine (HCQ) toxicity. The left eye (right column) shows normal findings across all imaging modalities.

A 41-year-old woman with a nine-year history of SLE had been taking 200 mg of HCQ daily (cumulative dose 657 g; 2.6 mg/kg ABW [200 mg/77 kg]). She denied renal disease, tamoxifen use, or other maculopathies. At her routine annual screening, swept-source OCT, fundus autofluorescence (FAF), and Humphrey visual fields were obtained. Best-corrected visual acuities were 20/30 OD and 20/22 OS. OCT of the right eye (Figure 1, top middle) showed parafoveal ellipsoid zone attenuation, and FAF (top left) revealed ring-shaped inferior hyperautofluorescence. Visual fields demonstrated scattered paracentral scotomas (top right). The left eye appeared normal on OCT (middle).

Three months earlier, she had experienced right-eye visual decline after her maintenance oral prednisolone (Solondo Tab^®^; YuHan Corp., Seoul, Republic of Korea) dose was increased from 5 mg to 10 mg daily for worsening SLE symptoms. At that visit, BCVA was 20/40 OD and 20/22 OS, and OCT revealed a serous macular detachment in the right eye (Figure 1, bottom left) that was absent on her prior annual OCT (bottom right). Following a diagnosis of steroid-induced central serous chorioretinopathy, her prednisolone was reduced back to 5 mg. At her subsequent HCQ screening three months later, the serous detachment had resolved. From the normal OCT image obtained one year ago, hydroxychloroquine retinopathy was very unlikely for explaining the photoreceptor defect. Comparison with historical and fellow-eye OCT confirmed CSCR as the cause of the photoreceptor loss, effectively excluding HCQ toxicity.

### 2.2. Case 2

A 37-year-old woman with a six-year history of SLE had been taking 300 mg of hydroxychloroquine daily (cumulative dose ~640 g; 6.1 mg/kg ABW [300 mg/49 kg]). She denied tamoxifen use or renal disease and had been on 7.5 mg of oral prednisolone (Solondo) daily for one year.

**Figure 2:**
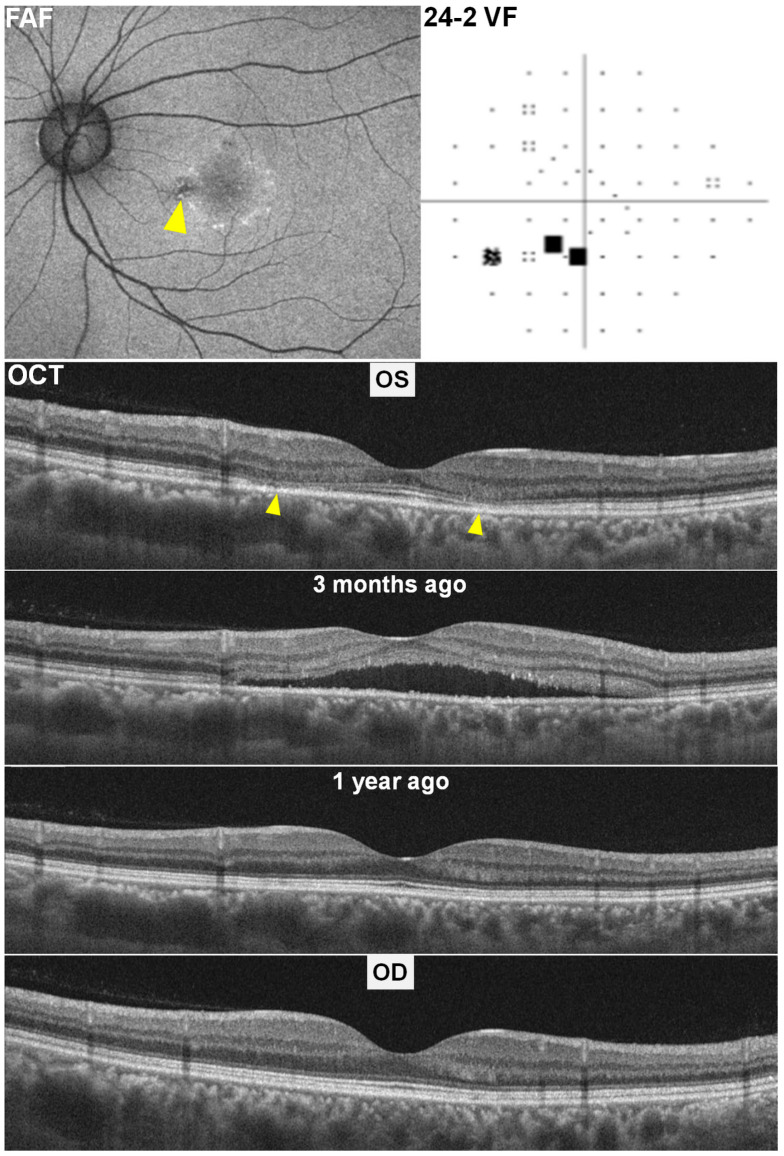
Multimodal imaging in Case 2 with steroid-induced central serous chorioretinopathy (CSCR) mimicking hydroxychloroquine toxicity. Top row: Left-eye fundus autofluorescence (FAF, left) shows a ring of hyperautofluorescence with scattered hyper- and hypoautofluorescent dots (arrowhead), and the 24-2 Humphrey visual field (24-2 VF; right) demonstrates a patchy inferotemporal scotoma. Bottom row: Swept-source optical coherence tomography (OCT) reveals parafoveal ellipsoid-zone loss (arrowheads) and outer retinal thinning (the “flying-saucer” sign) in the left eye. OCT from three months earlier shows shallow subretinal fluid, consistent with steroid-induced CSCR; OCT from one year prior shows normal outer retinal layers, confirming that the residual defects represent sequelae of CSCR. Right-eye OCT (bottom) is unremarkable.

At her routine HCQ screening, fundus autofluorescence (FAF) of the left eye (Figure 2, top left) showed a ring of hyperautofluorescence with scattered hypoautofluorescent dots, while swept-source OCT (top middle) revealed parafoveal ellipsoid-zone defects and outer retinal thinning (“flying saucer” sign). The fellow (right) eye appeared normal on OCT. Humphrey visual fields (24-2) in the left eye demonstrated a patchy inferotemporal scotoma.

Retrospective review of an OCT scan obtained from three months earlier (Figure 2, lower left) identified shallow subretinal fluid at the same parafoveal location, consistent with steroid-induced CSCR. After changing prednisolone (7.5 mg) to deflazacort (6 mg) (Calcort^®^, Handok Inc., Seoul, Republic of Korea), the subretinal fluid resolved at the subsequent examination performed for HCQ annual screening. The BCVA in the affected left eye had declined to 20/40 during the CSCR episode but improved to 20/25 following subretinal fluid resolution. An OCT from one year prior (Figure 2, lower right) showed intact outer retinal layers, confirming that the residual photoreceptor defects represented sequelae of resolved CSCR rather than HCQ toxicity.

### 2.3. Case 3

A 47-year-old female diagnosed with SLE was on long-term daily treatment with 200 mg of hydroxychloroquine for 25 years. Her body weight was 49 kg; daily dose/actual body weight (ABW) ratio was 4.1 mg/kg ABW. She had combined kidney disease with her estimated glomerulus filtration rate (eGFR) of 13 mL/min/1.73 m^2^. Concurrently, she had received 10 mg of prednisolone daily for two months.

**Figure 3:**
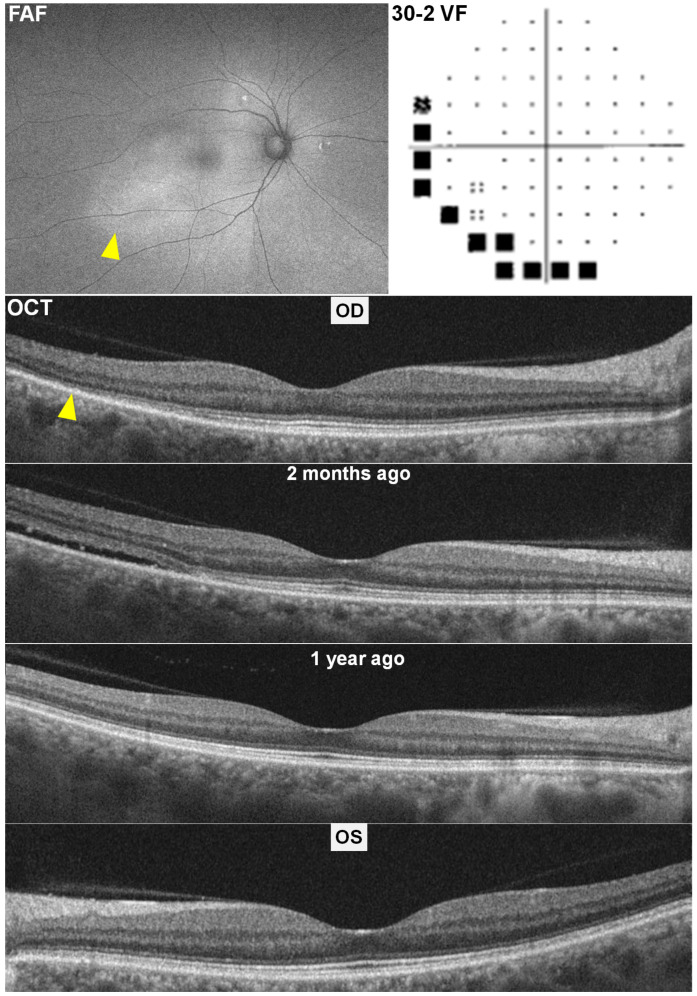
Multimodal imaging of Case 3 showing temporal pericentral photoreceptor defects on optical coherence tomography (OCT; arrowhead), corresponding temporal hyperautofluorescence on fundus autofluorescence (FAF; arrowhead), and inferior–nasal scotomas on 30-2 Humphrey visual field testing (30-2 VF). A prior OCT obtained two months earlier shows shallow subretinal fluid consistent with central serous chorioretinopathy (CSCR). OCT performed one year earlier and the OCT of the left eye are normal.

Multimodal screening with swept-source OCT, FAF, and HVF 30-2 revealed temporal pericentral photoreceptor defects in the right eye (Figure 3, arrowhead), corresponding inferotemporal hyperautofluorescence on FAF (arrowhead), and inferior–nasal circumferential scotomas on HVF. However, the left eye appeared structurally normal.

A retrospective review of prior OCT scans performed two months and one year earlier provided definitive diagnostic clarity. These images documented the presence of shallow subretinal fluid in the affected right eye two months ago, a finding consistent with CSCR. The BCVA in both eyes remained stable at 20/20 throughout the pre-, during-, and post-CSCR periods.

## 3. Literature Review of Potential Mimickers of Hydroxychloroquine Retinopathy

We performed a systematic literature search in PubMed and Embase (January 2000–May 2025) to identify reports of retinal conditions that can mimic HCQ retinopathy on multimodal imaging. Search terms included “hydroxychloroquine retinopathy”, “mimic”, “differential diagnosis”, and individual retinal disease names (e.g., “age-related macular degeneration”, “paraneoplastic retinopathy”, “macular dystrophy”, “maculopathy”, and “macular telangiectasia”). We included case reports, case series, and review articles in English. Titles and abstracts were screened for relevance; full texts were reviewed to extract clinical and imaging features, differentiating clues, and management implications. A summary of the identified mimicking conditions and their distinguishing features is presented in Table 1.

### 3.1. Paraneoplastic and Autoimmune Retinopathies

Paraneoplastic retinopathy (PNR) and autoimmune retinopathy (AIR) encompass a group of disorders in which circulating autoantibodies target retinal antigens, causing photoreceptor degeneration and RPE dysfunction [18,19,20]. In cancer-associated retinopathy (CAR), malignancies such as small-cell lung carcinoma can trigger anti-recoverin or anti-enolase antibodies that selectively damage photoreceptors [21]. Early in CAR, OCT may reveal focal or diffuse thinning of the outer nuclear layer (ONL) and disruption of the EZ, closely mimicking the parafoveal EZ attenuation seen in HCQ toxicity. FAF imaging can demonstrate mixed hyper- and hypoautofluorescent patches corresponding to RPE stress and photoreceptor loss, resembling the “bull’s-eye” pattern of HCQ retinopathy. Visual field testing often reveals ring scotomas in both conditions.

Key distinguishing features include the rate of progression, which is typically rapid in PNR/AIR (symptoms and imaging changes evolve over weeks to a few months), whereas HCQ toxicity usually develops over years of continuous exposure. Systemic evaluation often uncovers underlying malignancy or autoimmune markers; for instance, positive anti-recoverin titers help confirm CAR. Full-field electroretinography (ERG) in PNR often shows generalized photoreceptor dysfunction, with markedly reduced a- and b-wave amplitudes, while HCQ toxicity initially produces localized functional loss on multifocal ERG, sparing the foveal center longer.

### 3.2. Macular Dystrophies

Occult macular dystrophy (OMD), most commonly caused by mutations in the RP1L1 gene, presents with progressive central vision loss despite a near-normal fundus appearance [22]. OCT in OMD shows parafoveal EZ disruption and ONL thinning without overt pigmentary changes. FAF often remains normal until late stages, setting it apart from HCQ toxicity, where an early hyperautofluorescent ring may be evident. Visual acuity decline in OMD is disproportionate to structural findings, and the disease course is lifelong but slowly progressive over decades, in contrast to the dose-dependent acceleration seen with HCQ use. Genetic testing confirms the diagnosis, providing definitive differentiation.

Stargardt disease is the most common inherited macular dystrophy, affecting roughly 1 in 10,000 individuals, and is caused by biallelic or occasionally heterozygous mutations in the ABCA4 gene [23]. Clinically, it can sometimes mimic HCQ toxicity—particularly when a perifoveal ring of atrophy produces a “bull’s-eye” appearance—prompting investigation into ABCA4 variants among patients referred for HCQ retinopathy. Indeed, small series have identified heterozygous ABCA4 mutations in HCQ-suspected cases, suggesting reduced transporter function may predispose to outer retinal injury [24]. However, true Stargardt disease is distinguished by an earlier age of onset (typically in the first to third decade), characteristic yellow-orange pisciform flecks at the posterior pole with peripapillary sparing, and progressive geographic atrophy of the macula often without accompanying pigmentary changes. Fluorescein angiography in Stargardt reveals a “silent” choroid—widespread choroidal hypofluorescence—that is never seen in HCQ retinopathy, and OCT angiography demonstrates superficial capillary thinning rather than the parafoveal hyperautofluorescent ring and EZ attenuation/loss typical of HCQ toxicity [23].

Pattern dystrophies (e.g., butterfly-shaped, bifocal vitelliform) can generate lipofuscin-like deposits [25] leading to a mottled FAF appearance and outer retinal changes on OCT. These dystrophies frequently show distinct morphologies (fleck-like lesions, vitelliform material) and follow a benign, slowly progressive course unlinked to drug exposure. Genetic tests (e.g., BEST1, PRPH2, CTNNA1 mutations) confirm these hereditary disorders and exclude HCQ toxicity [26].

### 3.3. Macular Telangiectasia Type 2 (MacTel 2)

Macular telangiectasia type 2 is a bilateral neurodegenerative and vascular disease characterized by parafoveal telangiectatic capillaries, Müller cell loss, and photoreceptor degeneration [27]. In advanced MacTel 2, OCT may reveal inner-retinal cavitations and outer-retinal atrophy that can resemble the “flying saucer” sign or parafoveal thinning seen with HCQ toxicity. FAF can show a parafoveal hypoautofluorescent ring due to loss of the foveal luteal pigment and RPE changes, superficially similar to HCQ’s autofluorescent halo [28].

However, MacTel 2 has unique inner-retinal microstructural changes—low reflectivity spaces and right-angle venules—that are absent in HCQ retinopathy. Fluorescein angiography reveals late-phase parafoveal leakage from telangiectatic vessels. Moreover, MacTel 2 patients often retain good central visual acuity until late, whereas HCQ toxicity produces early paracentral scotomas and measurable reductions in multifocal ERG amplitude.

### 3.4. Age-Related Macular Degeneration (AMD)

Early non-neovascular AMD may present with drusenoid RPE pigment epithelial detachments and subretinal drusenoid deposits. On OCT, these can appear as hyperreflective elevations and overlying photoreceptor attenuation, superficially resembling early HCQ retinal thinning. FAF frequently demonstrates focal hyperautofluorescent drusen margins and hypoautofluorescent areas where RPE atrophy is present [29]. In contrast, HCQ toxicity produces a continuous parafoveal hyperautofluorescent ring rather than discrete drusen-related signals [30]. Color fundus photography in AMD reveals drusen and pigmentary changes absent in HCQ cases.

### 3.5. Solar and Photic Retinopathy

Exposure to intense light sources (solar eclipse, welding arcs) can induce focal photoreceptor damage, resulting in small central or paracentral EZ disruptions on OCT. FAF may show central hypoautofluorescence corresponding to RPE damage [31]. Visual field tests often reveal central scotomas in both solar retinopathy and HCQ toxicity. However, the acute onset tied to a known photic event, bilateral symmetric foveal lesions, and rapid partial recovery of photoreceptor integrity distinguish solar retinopathy from the chronic, progressive, and dose-related HCQ retinopathy.

### 3.6. Drug-Induced Mimickers Beyond HCQ

Other medications can produce retinal toxicity patterns similar to HCQ, including tamoxifen, thioridazine, and pentosan polysulfate (PPS). Tamoxifen retinopathy features refractile crystalline deposits in the macula, OCT-demonstrated photoreceptor loss, and patchy hypoautofluorescence. The presence of macular crystals on slit-lamp biomicroscopy and a history of tamoxifen exposure differentiates it from HCQ toxicity [32]. Thioridazine toxicity can cause diffuse RPE changes and outer retinal thinning; a history of high-dose phenothiazine use is the key distinguishing element. PPS maculopathy presents with pigment epithelium mottling, parafoveal atrophy, and a characteristic “fishing-net” pattern of hyper- and hypoautofluorescence on FAF [33]. OCT demonstrates focal loss of the ellipsoid zone and RPE attenuation that may mimic early HCQ changes. Distinguishing features include a history of long-term PPS use (often for interstitial cystitis), bilateral but asymmetric parafoveal atrophy, and a lack of the classic pericentral ring pattern seen in pericentral or mixed HCQ retinopathy. Ancillary testing—such as near-infrared reflectance, which often shows a spoke-wheel appearance in PPS maculopathy—further aids in differentiation.

### 3.7. Summary of Differentiating Strategies

To accurately distinguish HCQ retinopathy from its mimickers, clinicians should combine (1) detailed medication and exposure history, noting cumulative drug doses, timing of symptom onset relative to drug initiation or dose changes, and non-HCQ exposures (e.g., steroids, light sources); (2) bilateral and longitudinal imaging comparisons, seeking symmetry and progressive accumulation of atrophy in HCQ toxicity versus often unilateral or spontaneously resolving changes in mimickers; and (3) multimodal imaging cross-correlation, using FA and indocyanine green angiography to identify vascular leakage (e.g., MacTel 2) or choroidal hyperpermeability (e.g., steroid-induced CSCR) and FAF to differentiate drusen or pattern dystrophy signals from HCQ rings. Adjunctive testing includes full-field and multifocal ERG, antibody panels for paraneoplastic syndromes, and genetic sequencing for inherited dystrophies.

## 4. Discussion

The cases presented vividly illustrate the significant diagnostic challenge posed by macular pathologies in patients with SLE who are commonly receiving concurrent long-term HCQ and corticosteroids. Both steroid-induced CSCR and HCQ retinopathy can manifest with outer retinal changes and visual symptoms, leading to considerable diagnostic overlap. In our SLE cases, features mimicking parafoveal or pericentral HCQ toxicity were actually sequelae of resolved CSCR identified through the review of prior OCT scans.

Indeed, without reviewing prior images, the unilateral OCT abnormality could easily have been misread as HCQ retinopathy given each patient’s risk profile. All had been on HCQ for over five years, Patient 2’s daily dose exceeded 5 mg/kg ABW, and Patient 3 had significant renal impairment, which are considered as major risk factors [10,34,35]. In Patients 1 and 3, the screening physicians were unaware of the history of CSC, as it had been diagnosed by different ophthalmologists, and the photoreceptor defects were not specific enough to suggest prior CSC without reviewing earlier OCT images. Relying on current imaging findings from a single eye can be misleading, as residual photoreceptor changes from resolved CSCR may closely mimic those seen in HCQ toxicity. Comparison of both eyes is essential: HCQ retinopathy typically presents bilaterally and symmetrically [36], whereas CSCR typically presents unilaterally; however, bilateral involvement is relatively more common in older patients, with a prevalence of 50% in those aged 50 years or older compared to 28% in patients younger than 50 years [37,38].

The availability of prior OCT scans, clearly showing transient subretinal fluid that correlated with steroid use and tapering, was the key to diagnosing steroid-induced CSCR [39]. This underscores the critical importance of maintaining comprehensive patient records and obtaining baseline imaging to enable accurate comparison in the evaluation of HCQ retinopathy among at-risk patients. This also emphasizes a key principle in clinical ophthalmology: the indispensable value of baseline and serial imaging for patients on long-term medications with known ocular side effects. It supports shifting from a single point-in-time assessment to a longitudinal approach that incorporates historical comparisons.

Misdiagnosing resolved CSCR as HCQ retinopathy carries substantial risks. It could lead to the unnecessary cessation of hydroxychloroquine, a first-line, cornerstone therapy for managing SLE [17,40]. This could, in turn, result in severe SLE flares, increased systemic morbidity, and reduced quality of life. Conversely, failing to diagnose true HCQ toxicity (by mistaking it for CSCR) could delay the necessary discontinuation of the drug, leading to irreversible and progressive vision loss [10,41]. The clinical importance of differentiating these conditions is high, as both scenarios can impact both the patient’s long-term ocular health and the effective systemic management in SLE patients. This case may serve as a powerful cautionary tale, emphasizing that diagnostic precision is paramount to avoid significant adverse patient outcomes and optimize therapeutic strategies.

These cases highlight several key clinical insights. First, obtaining a thorough and accurate medication history, including exact dosages and durations of both hydroxychloroquine and corticosteroid use, particularly any recent dose increases, is essential. Second, establishing a routine baseline and serial, regular retinal imaging is strongly recommended for high-risk SLE patients on long-term HCQ and/or corticosteroids. The AAO guidelines recommend baseline imaging to rule out preexisting maculopathy in all long-term HCQ users, which also serves this broader diagnostic purpose [10]. Third, the importance of comparing current imaging with all prior examinations and the fellow eye cannot be overstated, as this often reveals subtle changes or resolves diagnostic uncertainty.

This report has some limitations. First, as a case series, the small number of patients limits the generalizability of the findings. Second, longer-term follow-up was not available, so the stability of the residual outer retinal changes over time could not be confirmed.

## 5. Conclusions

This case series unequivocally demonstrates that resolved steroid-induced CSCR in SLE patients typically on long-term hydroxychloroquine and concurrent systemic corticosteroid can present with outer retinal changes that closely mimic hydroxychloroquine toxicity. Accurate differential diagnosis necessitates a comprehensive clinical evaluation, a meticulous review of historical multimodal imaging, and careful comparison with the fellow eye. This approach is paramount to avoid misdiagnosis, prevent the unnecessary cessation of essential systemic therapy (such as hydroxychloroquine for SLE), and ensure appropriate, targeted management of these challenging patients.

## Figures and Tables

**Table 1:** Summary of hydroxychloroquine retinopathy mimickers reported in the literature and in this series.

Condition	Overlap with HCQ Retinopathy	Key Differentiators
Paraneoplastic/autoimmune retinopathies	Outer nuclear layer thinning, EZ disruption; ring scotomas on visual fields	Rapid onset/progression (weeks–months); positive antiretinal antibodies or malignancy; diffuse ERG changes
Macular dystrophy (e.g., occult macular dystrophy, Stargardt disease)	Parafoveal EZ disruption on OCT	Childhood/adolescent onset; foveal involvement; very slow progression; confirmatory genetic variant
Macular telangiectasia type 2 (MacTel 2)	Outer retinal cavitations; parafoveal FAF changes	Inner-retinal cavitations, right-angle venules on OCT; late-phase FA leakage; preserved central acuity longer
Age-related macular degeneration	Photoreceptor attenuation; FAF irregularities	Drusen lesions on color fundus; discrete FAF drusen margins vs. continuous HCQ ring
Solar retinopathy	Focal EZ disruptions; central scotomas on VF	Acute onset linked to known light exposure; bilateral symmetric central lesions; partial recovery over weeks
Tamoxifen/thioridazine/pentosan polysulfate toxicity	Photoreceptor loss on OCT; patchy FAF hypoautofluorescence	Presence of macular crystals (tamoxifen); high-dose phenothiazine history; lack of classic HCQ autofluorescent ring
Central serous chorioretinopathy	Photoreceptor defects ± RPE changes following resolution of subretinal fluid	Documentation of prior subretinal fluid in the affected area on historical OCT scans

ERG = electroretinogram; EZ = ellipsoid zone; FA = fluorescein angiography; FAF = fundus autofluorescence; HCQ = hydroxychloroquine; OCT = optical coherence tomography; RPE = retinal pigment epithelium; and VF = visual field.

## Data Availability

The original contributions presented in this study are included in the article. Further inquiries can be directed to the corresponding author.

## Abbreviations

The following abbreviations are used in this manuscript:

ABW	Actual body weight
BCVA	Best-corrected visual acuity
CSCR	Central serous chorioretinopathy
eGFR	Estimated glomerular filtration rate
ERG	Electroretinogram
FAF	Fundus autofluorescence
HCQ	Hydroxychloroquine
HVF	Humphrey visual field
OCT	Optical coherence tomography
PED	Pigment epithelial detachment
RPE	Retinal pigment epithelium
SLE	Systemic lupus erythematosus

## References

[1] Nicholson B., Noble J., Forooghian F., Meyerle C. (2013). Central serous chorioretinopathy: Update on pathophysiology and treatment. Surv. Ophthalmol..

[2] Daruich A., Matet A., Dirani A., Bousquet E., Zhao M., Farman N., Jaisser F., Behar-Cohen F. (2015). Central serous chorioretinopathy: Recent findings and new physiopathology hypothesis. Prog. Retin. Eye Res..

[3] Kido A., Miyake M., Tamura H., Hiragi S., Kimura T., Ohtera S., Takahashi A., Ooto S., Kawakami K., Kuroda T. (2022). Incidence of central serous chorioretinopathy (2011–2018): A nationwide population-based cohort study of Japan. Br. J. Ophthalmol..

[4] Kitzmann A.S., Pulido J.S., Diehl N.N., Hodge D.O., Burke J.P. (2008). The incidence of central serous chorioretinopathy in Olmsted County, Minnesota, 1980–2002. Ophthalmology.

[5] Rai B.B., Morley M.G., Bernstein P.S., Maddess T. (2020). Pattern of vitreo-retinal diseases at the national referral hospital in Bhutan: A retrospective, hospital-based study. BMC Ophthalmol..

[6] Haimovici R., Koh S., Gagnon D.R., Lehrfeld T., Wellik S., Central Serous Chorioretinopathy Case-Control Study G. (2004). Risk factors for central serous chorioretinopathy: A case-control study. Ophthalmology.

[7] Carvalho-Recchia C.A., Yannuzzi L.A., Negrao S., Spaide R.F., Freund K.B., Rodriguez-Coleman H., Lenharo M., Iida T. (2002). Corticosteroids and central serous chorioretinopathy. Ophthalmology.

[8] Marmor M.F., Carr R.E., Easterbrook M., Farjo A.A., Mieler W.F., American Academy of O. (2002). Recommendations on screening for chloroquine and hydroxychloroquine retinopathy: A report by the American Academy of Ophthalmology. Ophthalmology.

[9] Marmor M.F., Kellner U., Lai T.Y., Lyons J.S., Mieler W.F., American Academy of Ophthalmology (2011). Revised recommendations on screening for chloroquine and hydroxychloroquine retinopathy. Ophthalmology.

[10] Marmor M.F., Kellner U., Lai T.Y., Melles R.B., Mieler W.F., American Academy of Ophthalmology (2016). Recommendations on Screening for Chloroquine and Hydroxychloroquine Retinopathy (2016 Revision). Ophthalmology.

[11] Carter E.E., Barr S.G., Clarke A.E. (2016). The global burden of SLE: Prevalence, health disparities and socioeconomic impact. Nat. Rev. Rheumatol..

[12] Nikolaidou A., Gianni T., Sandali A., Toumasis P., Benekos K., Tsina E. (2025). Ocular manifestations of Juvenile Systemic Lupus Erythematosus: A systematic review. Eye.

[13] Silpa-archa S., Lee J.J., Foster C.S. (2016). Ocular manifestations in systemic lupus erythematosus. Br. J. Ophthalmol..

[14] Kwon H.Y., Ahn S.J. (2024). Advances and outlook for lupus retinopathy. Expert Rev. Ophthalmol..

[15] Canadian Hydroxychloroquine Study G. (1991). A randomized study of the effect of withdrawing hydroxychloroquine sulfate in systemic lupus erythematosus. N. Engl. J. Med..

[16] Fanouriakis A., Kostopoulou M., Alunno A., Aringer M., Bajema I., Boletis J.N., Cervera R., Doria A., Gordon C., Govoni M. (2019). 2019 update of the EULAR recommendations for the management of systemic lupus erythematosus. Ann. Rheum. Dis..

[17] Fanouriakis A., Kostopoulou M., Andersen J., Aringer M., Arnaud L., Bae S.C., Boletis J., Bruce I.N., Cervera R., Doria A. (2024). EULAR recommendations for the management of systemic lupus erythematosus: 2023 update. Ann. Rheum. Dis..

[18] Canamary A.M., Takahashi W.Y., Sallum J.M.F. (2018). Autoimmune retinopathy: A Review. Int. J. Retin. Vitr..

[19] Weppelmann T.A., Khalil S., Zafrullah N., Amir S., Margo C.E. (2022). Ocular Paraneoplastic Syndromes: A Critical Review of Diffuse Uveal Melanocytic Proliferation and Autoimmune Retinopathy. Cancer Control.

[20] Boeck K., Hofmann S., Klopfer M., Ian U., Schmidt T., Engst R., Thirkill C.E., Ring J. (1997). Melanoma-associated paraneoplastic retinopathy: Case report and review of the literature. Br. J. Dermatol..

[21] Brossard-Barbosa N., Dezard V., Margolin E. (2023). Treatment of Cancer-Associated Retinopathy: A Systematic Literature Review. Ophthalmol. Retina.

[22] Miyake Y., Tsunoda K. (2015). Occult macular dystrophy. Jpn. J. Ophthalmol..

[23] Heath Jeffery R.C., Chen F.K. (2021). Stargardt disease: Multimodal imaging: A review. Clin. Exp. Ophthalmol..

[24] Shroyer N.F., Lewis R.A., Lupski J.R. (2001). Analysis of the ABCR (ABCA4) gene in 4-aminoquinoline retinopathy: Is retinal toxicity by chloroquine and hydroxychloroquine related to Stargardt disease?. Am. J. Ophthalmol..

[25] Pinckers A. (1988). Patterned dystrophies of the retinal pigment epithelium. A review. Ophthalmic Paediatr. Genet..

[26] Tanner A., Chan H.W., Pulido J.S., Arno G., Ba-Abbad R., Jurkute N., Robson A.G., Egan C.A., Knight H., Calcagni A. (2021). Clinical and Genetic Findings in CTNNA1-Associated Macular Pattern Dystrophy. Ophthalmology.

[27] Chew E.Y., Peto T., Clemons T.E., Sallo F.B., Pauleikhoff D., Leung I., Jaffe G.J., Heeren T.F.C., Egan C.A., Charbel Issa P. (2023). Macular Telangiectasia Type 2: A Classification System Using MultiModal Imaging MacTel Project Report Number 10. Ophthalmol. Sci..

[28] Jones P., Kalra G., Al-Sheikh M., Chhablani J. (2024). Mimickers of hydroxychloroquine retinal toxicity. Clin. Exp. Ophthalmol..

[29] Holz F.G., Steinberg J.S., Gobel A., Fleckenstein M., Schmitz-Valckenberg S. (2015). Fundus autofluorescence imaging in dry AMD: 2014 Jules Gonin lecture of the Retina Research Foundation. Graefes Arch. Clin. Exp. Ophthalmol..

[30] Kellner U., Renner A.B., Tillack H. (2006). Fundus autofluorescence and mfERG for early detection of retinal alterations in patients using chloroquine/hydroxychloroquine. Invest. Ophthalmol. Vis. Sci..

[31] Comander J., Gardiner M., Loewenstein J. (2011). High-resolution optical coherence tomography findings in solar maculopathy and the differential diagnosis of outer retinal holes. Am. J. Ophthalmol..

[32] Tenney S., Oboh-Weilke A., Wagner D., Chen M.Y. (2024). Tamoxifen retinopathy: A comprehensive review. Surv. Ophthalmol..

[33] Lindeke-Myers A., Hanif A.M., Jain N. (2022). Pentosan polysulfate maculopathy. Surv. Ophthalmol..

[34] Jorge A.M., Melles R.B., Marmor M.F., Zhou B., Zhang Y., Choi H.K. (2024). Risk Factors for Hydroxychloroquine Retinopathy and Its Subtypes. JAMA Netw. Open.

[35] Melles R.B., Marmor M.F. (2014). The risk of toxic retinopathy in patients on long-term hydroxychloroquine therapy. JAMA Ophthalmol..

[36] Lee J.M., Kwon H.Y., Ahn S.J. (2024). Atypical Presentations of Hydroxychloroquine Retinopathy: A Case Series Study. J. Clin. Med..

[37] Spaide R.F., Campeas L., Haas A., Yannuzzi L.A., Fisher Y.L., Guyer D.R., Slakter J.S., Sorenson J.A., Orlock D.A. (1996). Central serous chorioretinopathy in younger and older adults. Ophthalmology.

[38] van Rijssen T.J., van Dijk E.H.C., Yzer S., Ohno-Matsui K., Keunen J.E.E., Schlingemann R.O., Sivaprasad S., Querques G., Downes S.M., Fauser S. (2019). Central serous chorioretinopathy: Towards an evidence-based treatment guideline. Prog. Retin. Eye Res..

[39] Nicholson B.P., Atchison E., Idris A.A., Bakri S.J. (2018). Central serous chorioretinopathy and glucocorticoids: An update on evidence for association. Surv. Ophthalmol..

[40] Alarcon G.S., McGwin G., Bertoli A.M., Fessler B.J., Calvo-Alen J., Bastian H.M., Vila L.M., Reveille J.D., Group L.S. (2007). Effect of hydroxychloroquine on the survival of patients with systemic lupus erythematosus: Data from LUMINA, a multiethnic US cohort (LUMINA L). Ann. Rheum. Dis..

[41] Ahn S.J., Seo E.J., Kim K.E., Kim Y.J., Lee B.R., Kim J.G., Yoon Y.H., Lee J.Y. (2021). Long-Term Progression of Pericentral Hydroxychloroquine Retinopathy. Ophthalmology.

